# Solar and wind power data from the Chinese State Grid Renewable Energy Generation Forecasting Competition

**DOI:** 10.1038/s41597-022-01696-6

**Published:** 2022-09-21

**Authors:** Yongbao Chen, Junjie Xu

**Affiliations:** 1 School of Energy and Power Engineering, University of Shanghai for Science and Technology, Shanghai, 200093 China; 2 Shanghai Key Laboratory of Multiphase Flow and Heat Transfer in Power Engineering, Shanghai, 200093 China

**Keywords:** Wind energy, Solar energy

## Abstract

Accurate solar and wind generation forecasting along with high renewable energy penetration in power grids throughout the world are crucial to the days-ahead power scheduling of energy systems. It is difficult to precisely forecast on-site power generation due to the intermittency and fluctuation characteristics of solar and wind energy. Solar and wind generation data from on-site sources are beneficial for the development of data-driven forecasting models. In this paper, an open dataset consisting of data collected from on-site renewable energy stations, including six wind farms and eight solar stations in China, is provided. Over two years (2019–2020), power generation and weather-related data were collected at 15-minute intervals. The dataset was used in the Renewable Energy Generation Forecasting Competition hosted by the Chinese State Grid in 2021. The process of data collection, data processing, and potential applications are described. The use of this dataset is promising for the development of data-driven forecasting models for renewable energy generation and the optimization of electricity demand response (DR) programs for the power grid.

## Abstract (summary)

**Table:** 

Measurement(s)	renewable energy generation
Technology Type(s)	supervisory control and data acquisition system
Sample Characteristic - Location	China

## Background & Summary

The usage of renewable energy is increasingly important to reduce carbon emissions and protect our environment. Currently, renewable energy penetration in the grid is increasing worldwide. The power supply must simultaneously match the demand; otherwise, power imbalance problems occur in the power grid. These problems hinder the continuous development of renewable energy^1^, and overgeneration problems occur^2,3^. As renewable energies such as solar energy and wind power are intermittent energy resources, it will be difficult for these energy sources to fully replace fossil energy in the foreseeable future. Energy storage and demand response (DR) are two promising technologies that can be utilized to alleviate power imbalance problems and provide more renewable energy in the power grid in the future^4^.

Despite implementing DR or designing an energy storage system, an accurate forecasting model for renewable energy generation is crucial to optimize the power system and allow more renewable energies to penetrate into the grid^5^. Without accurate and reliable forecasting of renewable energy generation, the maximum benefits from the energy management system cannot be realized. Usually, renewable energy generation forecasting can be categorized into four types based on the time horizon, i.e., very short term (less than 30 min), short term (30 min-6 h), medium term (6–24 h) and long term (1–7 d)^6^. However, unlike forecasting the electrical consumption of a building, which is generally regular, forecasting renewable energy generation is notoriously difficult due to energy generation variability, which, according to previous studies, is deeply influenced by meteorological conditions^7,8^. Data-driven models such as machine learning algorithms have been well recognized in the field of big data science to deduct nonlinear relationships between independent and dependent variables^9^. Therefore, researchers have spent much effort on developing machine learning models. Machine learning algorithms such as generative adversarial networks (GANs), convolutional neural networks (CNNs), long short-term memory (LSTM) and ensemble methods are widely used^8,10^. GANs have been considered the most efficient algorithm to capture the intermittency and fluctuation characteristics of wind and solar energy generation in recent years^11,12^. GANs is a promising architecture in renewable scenarios generation, owing to the ability to avoid complex feature extraction and cumbersome manual labeling process that are required in the conventional data-driven model^12^. Furthermore, GANs can effectively depict the inherent stochastic and dynamic characteristics of renewable resources with no need for statistical assumptions. All in all, GANs leverages the capabilities of deep learning and the power of data-driven techniques to address the difficulty of scenario generation.

**Fig. 1:**
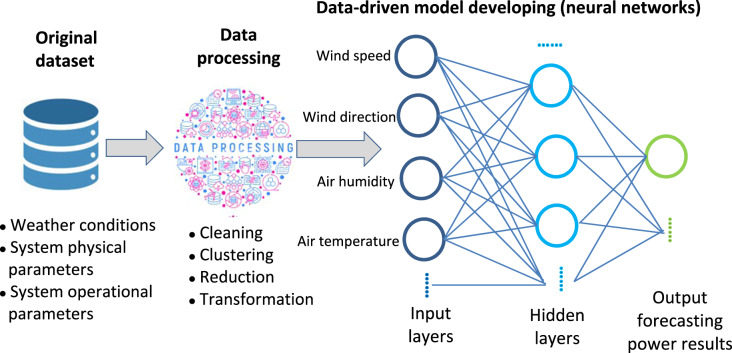
Flow diagram of data-driven model development process for wind energy forecasting.

The amount and quality of the dataset is the fundamental factor in the development of a data-driven forecasting model. Figure 1 shows the main diagram of developing a data-driven model for wind energy generation forecasting. Generally, there are two types of original datasets: simulated datasets and on-site collected datasets. The NREL Wind Integration Dataset is a widely used dataset^13^, and it provides simulated wind data from more than 126,000 land-based and offshore wind power production sites with a 2-km grid over the United States at a 5-min resolution. Datasets derived by analyzing satellite imagery are also common and effective. Through this method, a large-scale (i.e., city- or country-scale) dataset can be obtained. Simulated datasets are usually based on assumptions that are not always in accordance with real situations. On-site measurements are usually more accurate, and they are also more appropriate for the development of forecasting modes for a specific location. However, these data are difficult to collect. Agee *et al*. reported over six years of solar energy production data at a 1-hour resolution from a residential building (328 m^2^) in Virginia, USA^14^. Zhang *et al*. presented the global offshore wind turbine dataset^15^. There is a platform called OpenStreetMap that is used to recreate new versions of wind and solar installation datasets^16^. Solar radiation information is an indispensable parameter in analyzing solar generation. Jiang *et al*. presented a twelve-year (2007–2018) hourly dataset with 5-km resolution of surface and diffuse solar radiation in China^17^. Furthermore, more dataset repositories can be found in the review in^8^.

Although some solar and wind generation datasets have been made publicly available, few of them have focused on on-site wind farms and solar stations. Compared with simulated datasets, the on-site dataset is more meaningful for the development of a good generalization model. In developing a data-driven model to forecast renewable energy generation, feature variables such as wind speed and direction, solar irradiance and temperature are important variables used to train and validate the model. The motivation of this paper is to provide an on-site collected dataset for a better understanding of renewable energy generation characteristics, which are influenced by meteorological conditions and system parameters. Therefore, data-driven models can be developed using the dataset. This dataset was collected from six wind farms and eight solar stations in China. Based on this approach, solar and wind power forecasting models can be conveniently trained and validated.

## Methods

**Fig. 2:**
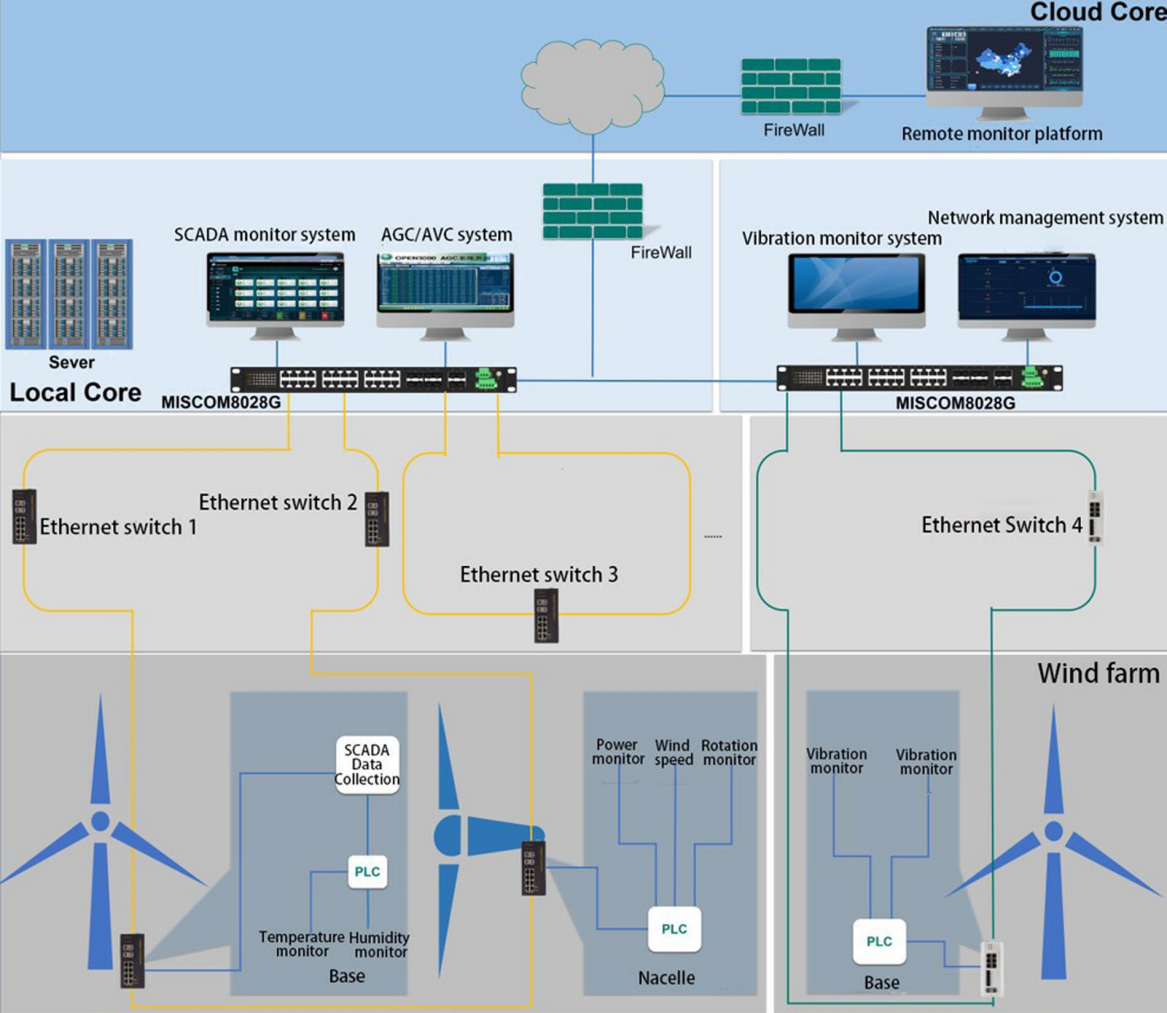
Sensor architecture and data collection process of the wind farms.

**Fig. 3:**
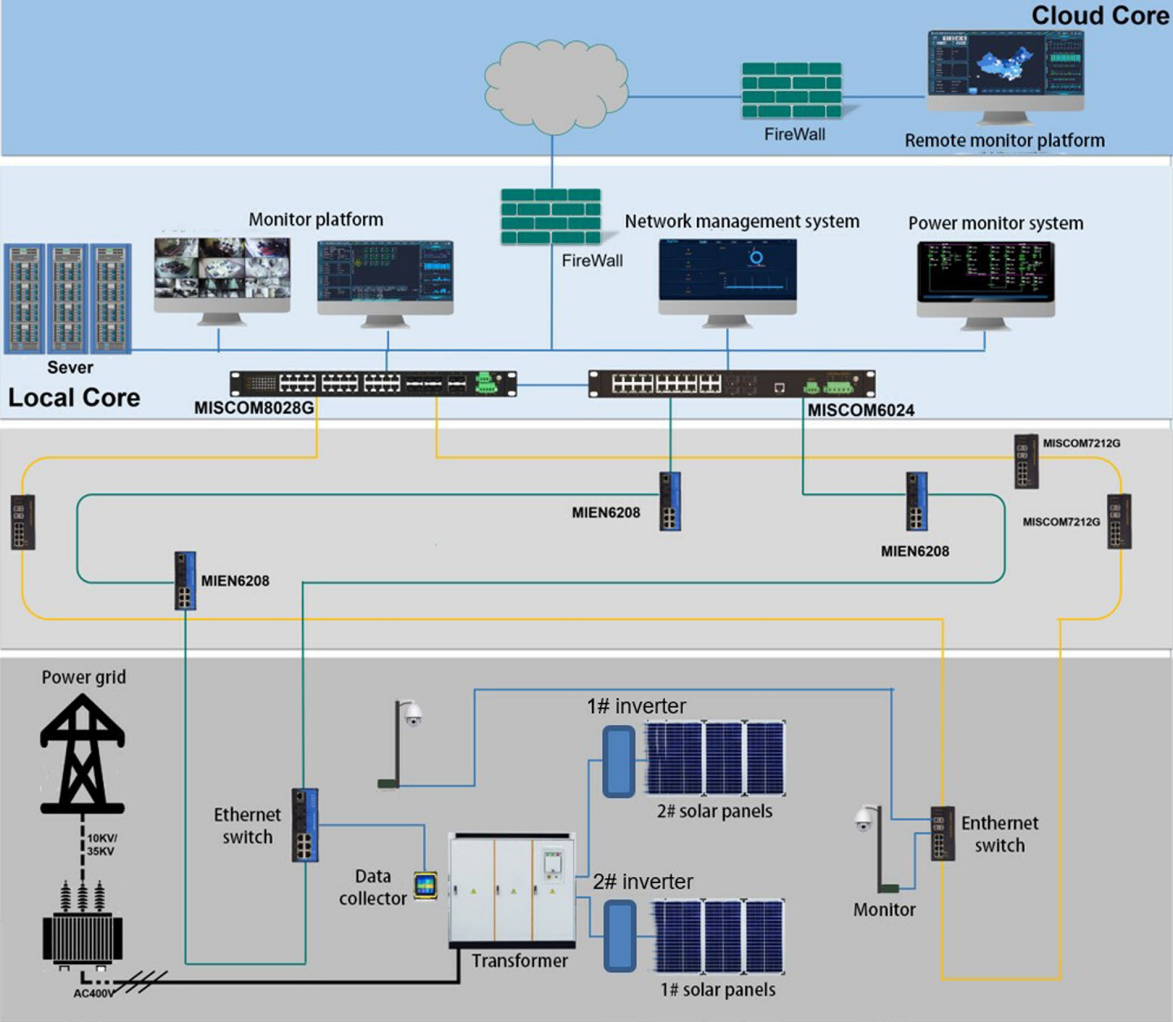
Sensor architecture and data collection process of the solar stations.

Wind farms and solar stations are generally equipped with a supervisory control and data acquisition (SCADA) system that connects hardware and software for monitoring, controlling and analyzing processes such as data visualization, alarm function, fault detection and emergency offload. A detailed introduction of the SCADA system can be found in^18^. The data of these six selected wind farms and eight solar stations were collected using SCADA systems. The facilities’ basic information and the nominal output capacity are listed in Tables 1, 4. The sensor architecture of the monitor systems for wind farms and solar stations are presented in Fig. 2 and Fig. 3, respectively. Data were accessed through the remote monitor platform and downloaded as.xlsx files by the authorized owner. The nominal power output capacity of these selected wind farms ranged from 36 MW to 200 MW, and the capacity of these selected eight solar stations ranged from 30 MW to 130 MW.

**Table 1:** Basic information on the wind turbines of each wind farm, which includes the wind turbine model and number and detailed information.

Wind farm name	Nominal generation output capacity (MW)	Wind turbine model	Detailed turbine information	Number of turbines
Farm site 1	75	GW1500/85	Capacity: 1500 kW	50
			Hub height: 85.0 m	
			Rotor diameter: 87.0 m	
			Website: https://en.wind-turbine-models.com/turbines/1201-goldwind-gw-87-1500	
	24	H93 L-2.0mw	Capacity: 2000 kW	12
			Hub height: 85.5 m	
			Rotor diameter: 93.0 m	
			Website: https://market.hzwindpower.com/?Service/Pro/Product24/2.html	
Farm site 2	200	GW3000/110	Capacity: 3000 kW	67
			Hub height: 120.0 m	
			Rotor diameter: 140.0 m	
			Website: https://en.wind-turbine-models.com/turbines/1738-goldwind-gw-140-3000	
Farm site 3	49.5	UP86-1500	Capacity: 1500 kW	33
			Hub height: 80.0 m	
			Rotor diameter: 86.0 m	
			Website: https://en.wind-turbine-models.com/turbines/292-united-power-up1500-86	
	49.5	UP82-1500	Capacity: 1500 kW	33
			Hub height: 80.0 m	
			Rotor diameter: 82.0 m	
			Website: https://en.wind-turbine-models.com/turbines/292-united-power-up1500-86	
Farm site 4	30	FD89A-1500	Capacity: 1500 kW	20
			Hub height: 85.0 m	
			Rotor diameter: 89.0 m	
			Website: https://en.wind-turbine-models.com/turbines/2224-dongfang-fd89-1500-geared	
	36	FD116A-2000	Capacity: 2000 kW	18
			Hub height: 90.0 m	
Farm site 5	36	FD116A-2000	Rotor diameter: 116.0 m	18
			Website: https://en.wind-turbine-models.com/turbines/2224-dongfang-fd89-1500-geared	
Farm site 6	96	XE72	Capacity: 2000 kW	48
			Hub height: 65.0 m	
			Rotor diameter: 70.7 m	
			Website: https://en.wind-turbine-models.com/turbines/616-xemc-ltd-xe72

To cover different climate zones and geographic locations, the selected solar stations and wind farm sites included areas in North, Central, and Northwest China, and the terrain included deserts, mountains and plains. It should be noted that all the original datasets were obtained and provided by a third-party, the Chinese State Grid, and the data collection process was out of the authors’ control.

## Data Records

In this section, the data types and the structure of the dataset, which can be downloaded from Figshare^19^ or GitHub (https://github.com/Bob05757/Renewable-energy-generation-input-feature-variables-analysis), are described. In the following subsections, the solar and wind data files are presented to guide users. There are two folders in the data repository; one is the folder that contains the original data with no data preprocessing, and the other folder contains data that was preprocessed based on the methods in *The processing of the missing data and outliers* subsection.

### Wind power generation

Wind power generation data are in the wind_farms folder, which includes six Microsoft Excel files. The real-time power generation and weather conditions are recorded in these files. The basic information about each wind farm is listed in Table 1.

In each Excel file, two years (2019–2020) of data, which included on-site weather conditions and power generation, with a time granularity of 15 minutes were recorded. Table 2 describes the meaning of the column headings. The wind speed at different height levels was recorded, and the speed at the wheel hub of the wind turbine was the most important factor for predicting power generation.

**Table 2:** Description of the feature variables.

Wind farm data file			Solar station data file		
Heading name	Shortened name	Description	Heading name	Shortened name	Description
Wind speed at height of *x* meters (m/s)	*WS_x*	The wind speed was recorded at *x* meters above the ground	Total solar irradiance (W/m^2^)	*TSI*	Solar power over all wavelengths per square meter
Wind direction at height of *x* meters (°)	*WD_x*	The wind direction was recorded at *x* meters above the ground	Direct normal irradiance (W/m^2^)	*DNI*	The amount of solar radiation received per square meter by a surface that is always held perpendicular to the rays
Air temperature (°C)	*Air_T*	Air dry-bulb temperature at *1.5* meters above the ground	Global horizontal irradiance (W/m^2^)	*GHI*	The total amount of shortwave radiation received by a surface horizontal to the ground
Atmosphere (hpa)	*Air_P*	Atmosphere at *1.5* meters above the ground	Air temperature (°C)	*Air_T*	Air dry-bulb temperature at *1.5* meters above the ground
Relative humidity (%)	*Air_H*	Air relative humidity at *1.5* meters above the ground	Atmosphere (hpa)	*Air_P*	Atmosphere at *1.5* meters above the ground
Power output(MW)	—	The total wind power generation	Relative humidity (%)	*Air_H*	Air relative humidity
			Power (MW)	—	The total solar power generation

While all variables are included, not all of them are required to develop a data-driven model.

The statistics of each wind farm can be seen in Table 3. The nominal wind generation capacity varied from 36 MW to 200 MW, and the average real output ranged from 6.7 MW to 72.7 MW. The wind speed at the height of the wheel hub varied from 0 m/s to 36.9 m/s, and the yearly average was approximately 6.0 m/s. The air temperature varied from −24.5 °C to 37.6 °C, and the yearly average was 8.5 °C. Weather conditions at different height levels showed a similar trend. Generally, the wind speed was seasonal, showing higher speeds during summertime and lower speeds during wintertime.

**Table 3:** Statistics of the wind farms.

Wind farm name	Statistics	Power output (MW)	Wind speed at the height of wheel hub (m/s)	Wind Direction at the height of wheel hub (°)	Air temperature at 1.5 meters above the ground (°C)	Relative humidity at 1.5 meters above the ground (%)
Farm site 1	Mean	23.4	6.4	217.0	8.5	37.6
	Minimum	0.0	0.0	0.0	−24.1	0.0
	Maximum	98.1	30.2	358.5	36.1	93.1
	Standard deviation	24.1	3.9	85.4	13.4	18.9
Farm site 2	Mean	72.7	7.5	206.8	8.7	33.4
	Minimum	0.0	0.0	0.0	−24.5	0.0
	Maximum	201.2	28.8	359.8	37.6	97.6
	Standard deviation	55.7	5.7	87.0	13.2	7.1
Farm site 3	Mean	18.1	4.0	179.1	17.4	58.5
	Minimum	0.0	0.0	0.0	−14.3	0.0
	Maximum	94.3	36.9	360.0	36.3	94.3
	Standard deviation	22.6	3.3	110.5	9.9	23.8
Farm site 4	Mean	17.4	5.5	147.3	13.8	80.7
	Minimum	0.0	0.0	0.0	−3.8	0.0
	Maximum	64.6	31.1	356.8	35.3	100.0
	Standard deviation	20.0	3.9	120.7	8.2	18.8
Farm site 5	Mean	6.7	4.7	184.9	13.6	69.9
	Minimum	0.0	0.0	0.0	−9.9	0.0
	Maximum	35.4	26.2	358.6	35.8	100.0
	Standard deviation	10.1	3.1	113.2	8.9	32.2
Farm site 6	Mean	28.8	8.1	94.0	21.2	78.6
	Minimum	0.0	0.0	0.0	0.0	0.0
	Maximum	114.4	23.8	360.0	37.1	99.4
	Standard deviation	28.0	3.8	91.2	6.4	10.9

The mean, minimum, maximum and standard deviation of each variable are presented.

### Solar energy generation

Solar power generation data are in the solar_stations folder, which includes eight Excel files. The weather condition data and real-time power generation data were recorded in these files. The power generation and PV panel information of each solar station are listed in Table 4. Similar to the wind generation dataset, two years (2019–2020) of data with a time granularity of 15 minutes were recorded. Table 2 describes the meaning of column headings. The nominal solar generation capacity varied from 30 MW to 130 MW, and the average real output ranged from 4.2 MW to 29.8 MW. The statistics of each solar station can be seen in Table 5.

**Table 4:** Power generation and PV panel information of each solar station, which includes the solar panel model and number and detailed information.

Solar station name	Nominal generation output capacity (MW)	PV panel model	Manufacturer and product websites	Number of PV panels installations
Station site 1	50	NA	NA	NA
Station site 2	130	NA	NA	NA
Station site 3	30	CS6U-325P	MFR: Canadian Solar Inc. Website: https://cn.csisolar.com/module/	27995
Station site 4	130	NA	NA	NA
Station site 5	110	JNMP60-255	MFR: Jinneng Clean Energy Technology Co.,Ltd. Website: https://www.jinergy.com/site/assembly/78	36828
Station site 6	35	SUN2000-50KTL-C	MFR: Huawei Technologies Co., Ltd. Website: https://support.huawei.com/enterprise/en/digital-power/sun2000-pid-7551590	703
Station site 7	30	NA	NA	60
Station site 8	0.93	HR-260P-18/Bbd	MFR: Hareon Solar Technology Co., Ltd. Links: out of service	3567
	1.92	HR-265P-18/Bbd		7234
	0.15	GCL-M6/60G280	MFR: Golden Concord Group System Integration Technology Co., Ltd. Website: https://www.gclsi.com/en/modules	541
	4.62	YL260P-29b	MFR: Yingli Green Energy Holding Co., Ltd. Website: http://www.solardesigntool.com/components/module-panel-solar/Yingli-Solar/3844/YL260P-29b/specification-data-sheet.html	17782
	6.96	JC260 M-24/Bb	MFR: ReneSola Co., Ltd. Website: http://www.solardesigntool.com/components/module-panel-solar/Renesola/2138/JC260 M-24-Bb/specification-data-sheet.html	26763
	1.56	CS6K-260P-PG	MFR: Canadian Solar Inc. Website: https://cn.csisolar.com/module/	5986
	6.47	CS6K-255P-PG		25383
	0.30	CS6K-250P-PG		1211
	2.32	TSM-260PC05A	MFR: Trina Solar Co., Ltd. Website: http://www.solardesigntool.com/components/module-panel-solar/Trina-Solar/1728/TSM-260-PC-PA05A/specification-data-sheet.html	8908
	4.24	SYP260P	MFR: Risen Energy Co., Ltd. Website: https://en.risenenergy.com/index.php?c=category&id=18	16326
	0.53	JMPV-HM6VBM2/60-340	MFR: Solargiga Energy holding Co., Ltd. Website: https://www.solargiga.com/productcenter/Component.html	1559

**Table 5:** Statistics of solar stations.

Solar station name	Statistics	Power output (MW)	Total solar irradiance (W/m2)	Direct normal irradiance (W/m2)	Global horizontal irradiance (W/m2)	Air temperature (°C)
Solar station site 1	Mean	9.7	266.4	93.3	67.7	13.1
	Minimum	0.0	0.0	0.0	0.0	−18.2
	Maximum	48.3	1359.0	980.0	989.0	41.2
	Standard deviation	13.7	368.0	200.8	111.2	14.3
Solar station site 2	Mean	19.6	169.6	122.4	78.3	13.7
	Minimum	0.0	0.0	0.0	0.0	−13.9
	Maximum	109.4	1041.9	751.8	561.8	40.5
	Standard deviation	28.0	248.4	179.2	117.6	12.1
Solar station site 3	Mean	5.2	81.1	111.1	66.3	—
	Minimum	0.0	0.0	0.0	0.0	—
	Maximum	29.9	1117.0	893.0	656.0	—
	Standard deviation	8.1	205.8	199.1	98.9	—
Solar station site 4	Mean	16.5	150.1	138.9	20.8	18.6
	Minimum	0.0	0.0	0.0	0.0	−5.3
	Maximum	114.7	1237.4	1010.3	151.0	49.8
	Standard deviation	27.5	253.5	210.6	31.5	10.3
Solar station site 5	Mean	14.5	164.3	147.9	115.0	17.8
	Minimum	0.0	0.0	0.0	0.0	−6.6
	Maximum	99.6	1467.0	1962.0	1208.0	39.5
	Standard deviation	23.9	273.5	234.9	203.1	9.6
Solar station site 6	Mean	6.4	244.1	216.0	54.1	20.6
	Minimum	0.0	0.0	0.0	0.0	2.9
	Maximum	31.2	1365.4	1179.8	296.2	36.7
	Standard deviation	9.2	355.9	338.0	69.4	5.8
Solar station site 7	Mean	5.4	206.8	—	—	—
	Minimum	0.0	0.0	—	—	—
	Maximum	29.8	3262.0	—	—	—
	Standard deviation	8.0	300.5	—	—	—
Solar station site 8	Mean	4.2	163.2	142.0	21.2	18.0
	Minimum	0.0	0.0	0.0	0.0	−8.0
	Maximum	29.4	1214.5	1056.7	157.9	47.6
	Standard deviation	6.5	245.4	213.5	31.9	8.6

The mean, minimum, maximum and standard deviation of each variable are presented.

## Technical Validation

In this section, the visualization of the data, which includes the processing of missing data, outliers, and correlation analysis of the influencing feature variables, is presented to clarify the data quality.

### The processing of the missing data and outliers

The missing data include variables that were zero, null, ‘NA’, ‘0.001’, ‘−99’, and ‘–’. The outliers included weather variables that remained unchanged over a long time, atmosphere values that were equal to zero, and the values that were unreasonably high or low. Table 6 shows the rate of outliers and missing data in the original dataset.

**Table 6:** Missing data and outlier rate of the dataset.

Wind farm data file			Solar station data file		
Farm site	Total sample size	Missing data and outliers’ rate	Station site	Total sample size	Missing data and outliers’ rate
Site 1	70176	1.58%	Site 1	70176	0.09%
Site 2		0.45%	Site 2		4.50%
Site 3		1.39%	Site 3	52608	78.25%
Site 4		3.25%	Site 4	70176	13.26%
Site 5		5.13%	Site 5		13.41%
Site 6		0.27%	Site 6		1.96%
			Site 7		4.48%
			Site 8	69408	6.15%

Missing data include variables that were zero, null, ‘NA’, ‘0.001’, ‘-99’, and ‘--’ in the data_original folder.

**Fig. 4:**
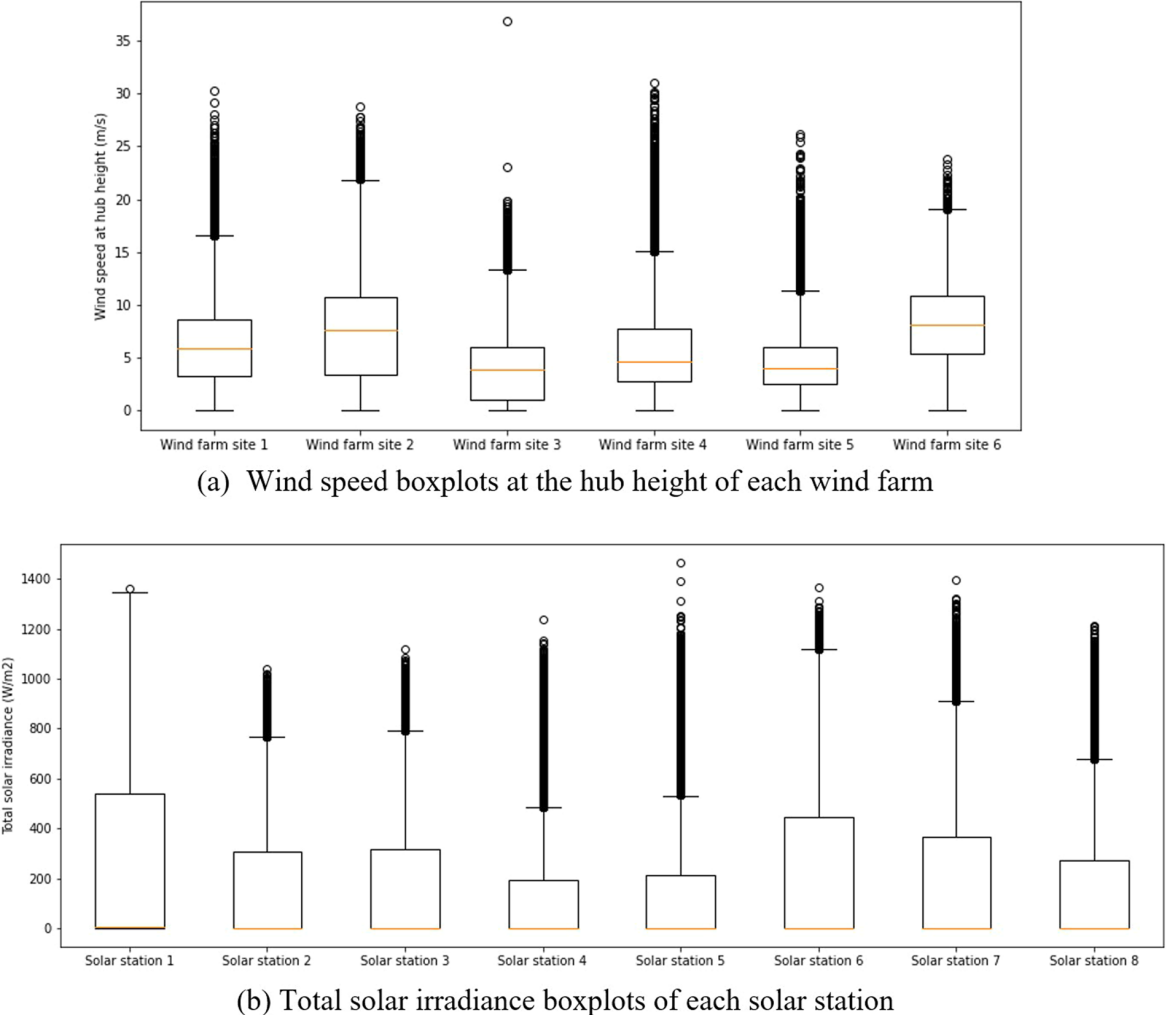
Boxplots of the key features of wind farms and solar stations. Before plotting these boxplots, the missing data, such as ‘-99’ and ‘null’, were dropped. Although there are several feature variables in the dataset, we selected the most important one to show the quartiles and outliers. In subplot (**a**), the wind speed at hub height is presented, and in subplot (**b**), the total solar irradiance is presented. The Jupyter notebook on the data processing and visualization can be found in the GitHub repository (https://github.com/Bob05757/Renewable-energy-generation-input-feature-variables-analysis).

There are many different approaches to preprocessing data, and users can use any appropriate methods that they are familiar with or proficient in. We suggest an upward/downward completion or a linear interpolation approach for the data samples where small steps (e.g., less than 10 steps) are missing. A moving average method can be considered when intermittent time steps (e.g., less than 100 steps) are missing; however, for long-term (e.g., more than 100 steps) missing data cases, the removal of these samples is recommended. In addition, the on-site dataset should not be adopted if the missing data rate is larger than a specific rate (i.e., 20%) of the total dataset; for example, at solar station site 3, most of the total solar irradiance points were outliers after August 1^st^, 2019. Figure 4 shows the boxplot of one key feature variable of wind and solar generation (missing data points were dropped before plotting the boxplot). The outliers can be seen in this figure. We provided both the original and processed dataset in the repository so that users can process the missing data and outliers using their own rules or use the processed dataset directly. It is worth noting that we only processed missing data such as ‘NA’, ‘0.001’, ‘-99’, and ‘--’ in the data files of data_processed folder, and the used approach was the simplest upward/downward completion. The outliers shown in Fig. 4 could be removed or not according to the data user themselves because these data points are classified as outliers by a specific criterion that the data is outside 1.5 times the interquartile range (IQR) including above the upper quartile (Q3 + 1.5*IQR) and below the lower quartile (Q1-1.5*IQR). Owing to the fluctuated characteristics of renewable energy, actually, some outliers in Fig. 4 could be a meaningful data point for developing a data-driven forecasting model.

### Correlation analysis

**Fig. 5:**
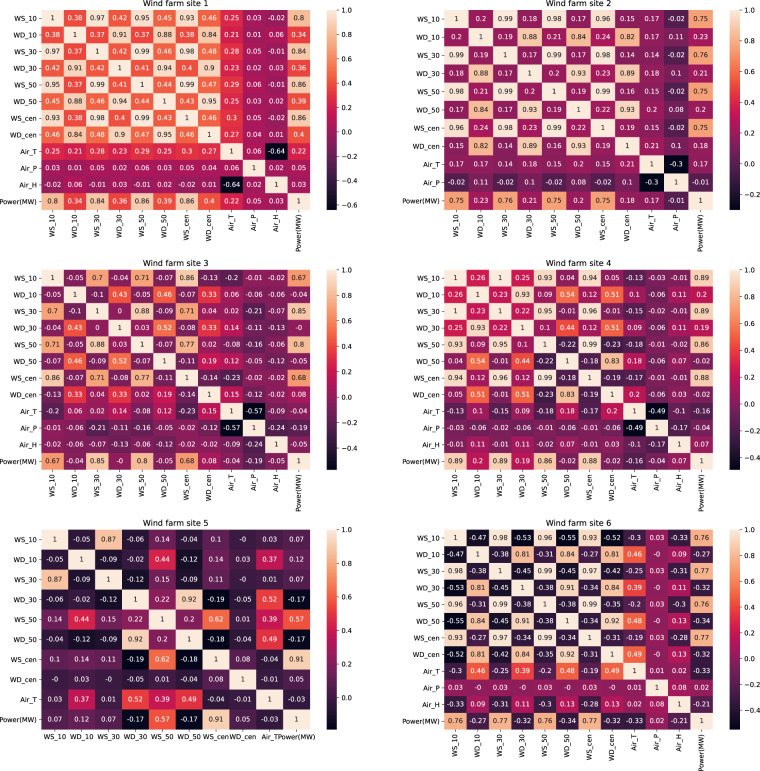
Pearson correlation coefficient of different variables of the wind farms. WS_*x (*i.e.*, wind speed at different* heights) has the highest PCC with respect to power. The hub height is different for each model of the wind turbine, so WS_cen represents different heights. The hub heights are 85 m, 120 m, 80 m, 85 m/90 m, 90 m, and 65 m for wind farm sites 1, 2, 3, 4, 5, and 6, respectively.

**Fig. 6:**
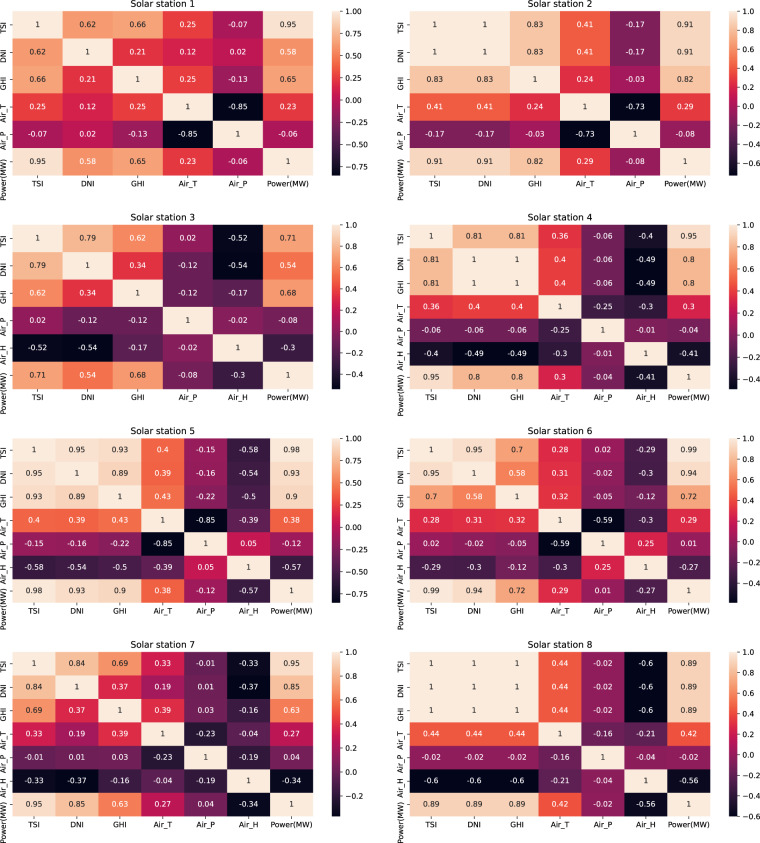
Pearson correlation coefficient of different variables of the solar stations. Generally, TSI has the highest PCC with respect to power.

In developing a data-driven forecasting model, selecting the proper input feature variables can improve the forecasting performance; therefore, correlation analysis is important for selecting the variables. Wind speed and solar radiation are the most important factors for generating wind and solar power, respectively. The Pearson correlation coefficient (PCC) is a measure of linear correlation between two sets of data. We found that the PCC between wind speed and power output in the wind dataset is much higher than other parameters, such as temperature and pressure (see Fig. 5). Similarly, in the solar dataset, total solar irradiance has the highest PCC with the power output, as shown in Fig. 6.

## Usage Notes

The data preprocessing methods for the missing data and outliers impact the forecasting performance of machine learning models. The dataset was used for the Chinese State Grid Renewable Energy Generation Forecasting Competition. On-site weather conditions such as wind speed, wind direction, and solar radiation are the main input feature variables that influence the generation of power. For the wind generation power forecasting case, wind speed is the main factor. For the solar energy generation case, solar radiation variables are the main factors. Many machine learning algorithms, such as GANs, LightGBM, SVM, random forest, CNNs, and LSTM, can be developed using this dataset to predict wind and solar energy generation in the short term in the future (e.g., one day or one week). It is worth noting that forecasting weather data is required when the developed model is used to perform forecasting tasks.

The selection of the input feature variables is important for developing a model. Generally, more dimensions of input feature variables could improve the forecasting performance owing to more information being taken into consideration. However, some variables are highly correlated, such as wind speed, at different height levels.

**Fig. 7:**
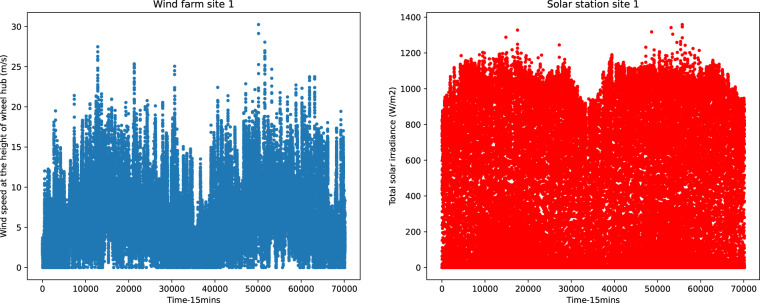
Seasonal trends of the main feature variable.

In the process of training and validating our model, we found that the implementation of data classification technology can improve forecasting accuracy. As shown in Fig. 7, the wind speed and solar radiation change seasonally. Several classification methods are suggested, including seasonal classification, classification by wind speed, and classification by the intensity of solar radiation. When we make the classification, each classification label should have a similar sample size. Table 7 shows one of the classifications by wind speed examples in the case of forecasting wind power generation.

**Table 7:** An example of classification by wind speed.

Classification label	Wind speed *v* tiers (m/s)
0	*v* ≤ 2.5
1	2.5 < *v* ≤ 5.0
2	5.0 < *v* ≤ 7.0
3	7.0 < *v* ≤ 9.0
4	9.0 < *v* ≤ 11.0
5	11.0 < *v*

Another application of this dataset is the beneficial implementation of DR programs in the grid. For power grids, especially a distributed energy system, renewable energy is intermittent, so the demand side should be coordinately managed with power generation. With the forecasting of days-ahead renewable energy generation, energy management and control systems can be further optimized.

## Data Availability

All the code and processing scripts used to produce the results of this paper were written in Python, Jupyter lab. Links to scripts and data for analysis can be found in the GitHub repository (https://github.com/Bob05757/Renewable-energy-generation-input-feature-variables-analysis).
